# Induced Pluripotent Stem Cells and CRISPR-Cas9 Innovations for Treating Alpha-1 Antitrypsin Deficiency and Glycogen Storage Diseases

**DOI:** 10.3390/cells13121052

**Published:** 2024-06-18

**Authors:** Colin Walsh, Sha Jin

**Affiliations:** Department of Biomedical Engineering, Thomas J. Watson College of Engineering and Applied Sciences, State University of New York at Binghamton, Binghamton, NY 13902, USA

**Keywords:** induced pluripotent stem cells, CRISPR-Cas9, alpha-1 antitrypsin deficiency, glycogen storage disease, gene therapy, metabolic disorders, therapeutic modeling

## Abstract

Human induced pluripotent stem cell (iPSC) and CRISPR-Cas9 gene-editing technologies have become powerful tools in disease modeling and treatment. By harnessing recent biotechnological advancements, this review aims to equip researchers and clinicians with a comprehensive and updated understanding of the evolving treatment landscape for metabolic and genetic disorders, highlighting how iPSCs provide a unique platform for detailed pathological modeling and pharmacological testing, driving forward precision medicine and drug discovery. Concurrently, CRISPR-Cas9 offers unprecedented precision in gene correction, presenting potential curative therapies that move beyond symptomatic treatment. Therefore, this review examines the transformative role of iPSC technology and CRISPR-Cas9 gene editing in addressing metabolic and genetic disorders such as alpha-1 antitrypsin deficiency (A1AD) and glycogen storage disease (GSD), which significantly impact liver and pulmonary health and pose substantial challenges in clinical management. In addition, this review discusses significant achievements alongside persistent challenges such as technical limitations, ethical concerns, and regulatory hurdles. Future directions, including innovations in gene-editing accuracy and therapeutic delivery systems, are emphasized for next-generation therapies that leverage the full potential of iPSC and CRISPR-Cas9 technologies.

## 1. Introduction

Metabolic and genetic disorders, characterized by disturbances in metabolic processes due to defects in the genes that control these processes, represent a significant area of clinical and research concern. Disorders such as alpha-1 antitrypsin deficiency (A1AD) and glycogen storage disease type I (GSDI) impair quality of life and pose substantial burdens due to their chronic nature and the complexity of their management. These conditions are primarily inherited and can lead to severe liver and pulmonary diseases, among other complications. Understanding these disorders at a molecular level is crucial for developing effective treatments [1,2]. Recent advances in biotechnology, mainly through the development of induced pluripotent stem cells (iPSCs) and CRISPR-Cas9 gene-editing technologies, have revolutionized the approach toward understanding and treating metabolic and genetic disorders. iPSC technology allows for the derivation of patient-specific pluripotent cells that can be differentiated into various cell types, providing a unique model system to study disease mechanisms and test therapeutic interventions in a patient-specific manner. This technology has shown significant potential in drug discovery and personalized medicine, offering insights into the pathophysiology of complex diseases [3,4]. CRISPR-Cas9, on the other hand, provides a robust, precise, and relatively simple method for gene editing that can correct genetic defects at the genome level. This approach has been applied successfully in correcting mutations responsible for diseases like A1AD and GSDI, offering hope for curative therapies that address the root cause of the diseases rather than just managing symptoms [5,6]. This review aims to assess the current state-of-the-art research and the future directions of A1AD and GSDI, particularly in the context of iPSC and CRISPR-Cas9 technologies. This review interprets recent findings that utilize these technologies to model and treat complex disorders. By examining the latest advancements and challenges, this review seeks to highlight the potential of iPSCs and CRISPR-Cas9 as tools for fundamental research and as pivotal elements in the translational pathway toward clinical applications. Overall, this review provides a comprehensive and updated overview that can aid researchers and clinicians in understanding the evolving landscape of genetic and metabolic disorder treatment for further development of innovative therapeutic solutions in the near future.

## 2. Overview of A1AD and GSDI

### 2.1. Genetic Basis and Clinical Manifestations of A1AD

A1AD is primarily caused by mutations in the *SERPINA1* gene, which encodes the alpha-1 antitrypsin (A1AT) protein, crucial for inhibiting proteases involved in inflammatory responses. The most common deleterious mutations result in a misfolded form of A1AT that accumulates in the hepatocytes, as summarized in Table 1. This accumulation diminishes the availability of A1AT in the bloodstream as well as impairing its protease inhibition function, which is critical for lung tissue protection. The deficiency of functional A1AT in the blood leads to an enzymatic imbalance that promotes unchecked proteolytic activity, resulting in tissue damage, primarily in the lungs [7,8]. Clinically, A1AD is associated with a spectrum of liver and pulmonary diseases. The pulmonary complications typically include early-onset emphysema and chronic obstructive pulmonary disease (COPD), which can manifest in individuals between 20 and 50 years of age. As shown in Table 1, liver disease varies from mild enzyme elevations to more severe conditions such as cirrhosis and hepatocellular carcinoma, observable even in childhood. These manifestations underscore the critical role of A1AT in maintaining the protease–antiprotease balance for lung and liver health [7,8].

### 2.2. Genetic Basis and Clinical Manifestations of GSDI

GSDI is predominantly caused by mutations in the glucose-6-phosphatase-α (*G6PC*) gene for GSDIa or the solute carrier family 37 (glucose-6-phosphate transporter) member 4 (*SLC37A4*) gene for GSDIb; both are pivotal for the proper function of glucose-6-phosphatase. As detailed in Table 1, these mutations lead to deficient activity of this enzyme, which is essential for the final steps of glycogenolysis and gluconeogenesis—the metabolic pathways that ensure glucose release into the bloodstream. The resultant enzymatic deficiency triggers an excessive accumulation of glycogen and fat in liver and kidney cells, leading to significant disruptions in normal cellular functions and metabolic homeostasis [8,9]. The clinical manifestations of GSDI, as highlighted in Table 1, typically include severe hypoglycemia, hepatomegaly due to glycogen accumulation, growth retardation, and metabolic acidosis. Patients also experience kidney disease, manifested as nephromegaly, which can progress to renal insufficiency. These symptoms are indicative of the disease’s profound impact on liver and kidney function, emphasizing the importance of early diagnosis and management to prevent long-term complications [8,9].

## 3. iPSCs for Modeling A1AD and GSDI 

Recent advancements in iPSC technology have significantly enhanced the efficiency of reprogramming and the safety of these cells for clinical applications, as well as the creation of disease-specific iPSC lines for diverse disease modeling and drug discovery [10,11,12,13]. Patient-specific iPSC lines have proven essential in recapitulating the pathobiology of targeted tissues or organs, such as the pancreas, heart, brain, and liver, offering crucial insights into complex diseases [13,14,15]. Significant achievements in using iPSC-derived models to explore the pathophysiology of A1AD and GSDI are highlighted in Table 2. For A1AD, iPSCs have been differentiated into hepatocyte-like cells, providing a dynamic model to study the accumulation and pathophysiology of alpha-1 antitrypsin (A1AT) within the liver [16,17]. These models facilitate an in-depth understanding of disease progression and response to therapies, closely mimicking the liver’s cellular environment. This close simulation allows for detailed observations of A1AT protein behavior and its impacts on liver function. In the case of GSDI, iPSCs have been instrumental in creating liver and kidney cell models that replicate the disease’s metabolic effects, particularly the dysfunctional processes of glycogenolysis and gluconeogenesis [9,18]. Three iPSC lines have been generated through reprogramming skin fibroblasts from three glycogen storage disease type III (GSDIII) patients [19]. These patient-derived iPSC lines are expected to be pivotal platforms for modeling GSDIII in vitro to study its pathological mechanisms and potential treatments. They can also be used for testing gene-editing tools and new pharmacological agents, enhancing the development of targeted therapeutic strategies.

### 3.1. Discoveries about A1AD Disease Mechanisms and Potential Therapies

While modeling liver diseases using iPSC-derived liver cells has been extensively studied since the invention of iPSC technology [20], recent advances in disease modeling using iPSC and CRISPR-Cas9 technologies have substantially enhanced our understanding of A1AD and opened new avenues for therapeutic discovery. This section summarizes pivotal findings from several current studies that employed patient-derived iPSCs and advanced genome-editing technology to investigate the pathophysiology of A1AD and explore innovative treatment strategies.

Recent developments in iPSC technology have focused on improving the efficiency and safety of reprogramming methods. Traditional reprogramming involves using four transcription factors—OCT4, SOX2, KLF4, and MYC [21]. However, newer techniques have emerged that utilize alternative transcription factors and small molecules to enhance reprogramming efficiency and reduce the risk of oncogenic transformation. For instance, Hou et al. demonstrated that combining small molecules could replace the traditional factors to induce pluripotency in mouse somatic cells, highlighting a significant advancement in the field [22]. Similarly, studies by Rais et al. and Shi et al. explored the use of small-molecule-based platforms and alternative transcription factors, which have been shown to improve the safety profile and efficiency of iPSC generation [23,24]. These advancements increase the potential applications of iPSCs in disease modeling and therapeutic development and reduce the possible risks associated with traditional reprogramming methods.

Additionally, advancements in CRISPR-Cas9 technology have expanded the possibilities for gene editing. The development of variant Cas9 proteins that enable single-base editing and avoid double-strand break events, such as Cas9 nickase (nCas9) and catalytically inactive Cas9 proteins (dCas9, dCas12a, dCas13b), have improved the precision and safety of gene editing [25]. Base-editing technology, including cytosine base editors and adenine base editors, allows for the precise modification of single base pairs at specific loci, further enhancing the accuracy of gene corrections [26]. For instance, Werder et al. used adenine base editors to correct the G-to-A point mutation in the *SERPINA1* gene in patient-derived iPSCs, leading to a significant decrease in polymerized A1AT within hepatocytes, showcasing a promising direction for gene therapy in A1AD [27]. Tafaleng et al. provided foundational insights into the personalized variations in liver disease caused by A1AD using patient-specific iPSC-derived hepatocytes. By reprogramming skin fibroblasts from A1AD patients, their study successfully differentiated these iPSCs into hepatocytes that recapitulated the liver disease phenotype, including the accumulation of misfolded A1AT protein. The study highlighted the utility of iPSC-derived hepatocytes in modeling disease severity and progression, underscoring the cellular mechanisms that contribute to liver pathology in A1AD patients [28,29]. Kaserman et al. expanded on this model by utilizing iPSC-derived hepatocytes from A1AD patients to discover potential therapeutic targets. Their work specifically focused on screening small molecules that could reduce the hepatic accumulation of defective A1AT and mitigate the associated liver damage. This study demonstrated the practical application of iPSC models in therapeutic discovery and identified candidate compounds that could potentially be repurposed for A1AD treatment [29]. Wilson et al. reported on the emergence of a stage-dependent liver disease signature by directing the differentiation of A1AD-deficient iPSCs [30]. Their detailed analysis of gene expression profiles throughout different stages of hepatocyte differentiation illuminated how A1AT mutation influences liver development and function. The study used a comprehensive array of biomarkers to assess disease-specific alterations, providing a deeper understanding of the developmental timeline of liver pathology in A1AD [30]. Furthermore, some studies collectively underscore the potential of iPSC technology in elucidating disease mechanisms, identifying novel therapeutic targets, and testing new treatment modalities [20,31]. These studies highlight the diverse applications of iPSC-derived models in replicating disease pathology, investigating genetic interventions, and exploring drug efficacy and safety. The ability to model A1AD in vitro using patient-derived iPSCs enhances our understanding of the disease and paves the way for personalized medicine approaches in managing and potentially curing A1AD in the future.

### 3.2. Advancements in Understanding Metabolic Impacts and Therapeutic Targets for GSDI

Research using iPSC-derived models of GSDI has provided breakthrough insights into the metabolic challenges posed by the disease, especially concerning the regulation of glucose production by the liver. These models are essential for the in vitro testing of new pharmacological agents that could assist in managing blood sugar levels and reducing glycogen accumulation in tissues [18]. These models have also facilitated the exploration of targeted gene therapies that aim to correct the underlying genetic defects of GSDI, offering potential advancements in treatment protocols that could directly address the enzymatic deficiencies.

Katagami et al. established a human iPSC line, BRCi009-A, from a GSDIa patient harboring a mutation in the *G6PC* gene, which is crucial for catalyzing the final step in gluconeogenesis and glycogenolysis [32]. These iPSCs were differentiated into hepatocytes to study the defective glucose release characteristic of GSDIa. The differentiated hepatocytes exhibited decreased G6PC enzyme activity and recapitulated the patient’s metabolic phenotype, including disrupted glucose homeostasis and excessive glycogen accumulation [32]. This model is invaluable for testing therapeutic interventions to restore normal glucose levels in GSDI patients.

In a related study, researchers generated iPSCs from a GSDIb patient, which were differentiated into hepatocytes and neutrophils [33]. Their study uniquely highlighted the dual cellular impact of G6PT deficiency, affecting glucose metabolism and neutrophil function. The iPSC-derived hepatocytes displayed critical metabolic disturbances such as enhanced glycogen storage and dysregulated glucose production, mirroring the hepatic symptoms of GSDIb. Furthermore, the neutrophils derived from these iPSCs exhibited functional deficiencies, providing insights into the immune dysfunctions observed in GSDIb patients [33].

Additional studies have leveraged iPSC and CRISPR-Cas9 technologies to model related glycogen storage diseases, offering broader implications for the field. For instance, Rossiaud et al. created a disease model of GSDIII using CRISPR-Cas9 to edit human iPSCs [34]. Their research underscored the potential of iPSCs in modeling various glycogen storage diseases and developing targeted gene therapy approaches. Similarly, Naito et al. introduced a novel variant, p.Ile694Asn, into iPSCs derived from a healthy donor to study GSD4, revealing reduced GBE1 activity and increased polyglucosan body formation in hepatocytes and cardiomyocytes [35].

Kishnani et al. summarized the development of gene therapy for glycogen storage diseases, including GSDI, by targeting the genetic mutations causing enzyme deficiencies [36]. These studies underscore the versatility and efficacy of iPSC models in advancing our understanding of GSDI. They provide insights into the disease mechanisms and facilitate the development of novel therapeutic strategies that could lead to effective treatments or even cures for these challenging metabolic disorders. As iPSC and CRISPR-Cas9 technologies continue to evolve, they promise to significantly advance theoretical knowledge and clinical applications for managing and potentially curing GSDI.

**Table 2:** Key achievements and technical challenges in iPSC research for A1AD and GSDI disorders.

Aspect	A1AD Achievements	A1AD Challenges	GSDI Achievements	GSDI Challenges	References
Disease Modeling	Developed iPSC-derived hepatocyte models to study A1AT accumulation and liver fibrosis.	Difficulty in fully replicating the liver environment and its complex interactions in vitro.	Created detailed models of liver and kidney cells to study metabolic dysfunctions and glycogen accumulation.	Challenges in replicating exact physiological conditions of glucose metabolism in vitro.	[16,17,20,28]
Gene Therapy	Utilized CRISPR-Cas9 to correct *SERPINA1* gene mutations directly in iPSCs.	Ensuring long-term stability and integration of corrected genes without off-target effects.	Tested gene-editing tools to correct genetic defects in the *G6PC* and *SLC37A4* genes.	Managing off-target effects and ensuring precise gene correction in all affected cells.	[9,36,37,38,39,40]
Drug Testing	iPSC models used for screening potential therapeutic compounds that can alleviate liver fibrosis.	Variability in drug responses due to patient-specific iPSC differences.	Enabled preclinical testing of new pharmacological agents to manage glycogen storage and enhance glucose release.	Difficulty in predicting clinical efficacy based on iPSC-derived model results.	[1,11,16,18]
Pathophysiological Insights	Revealed mechanisms of inflammatory response and oxidative stress due to A1AT deficiency.	Requires more comprehensive models that include immune and other systemic interactions.	Provided insights into abnormal glucose-6-phosphatase activity and its systemic effects.	Requires deeper understanding of long-term disease progression and secondary complications.	[8,9,41,42]
Therapeutic Development	Opened avenues for developing targeted therapies that can be personalized based on the patient’s genetic profile.	Development and regulatory challenges in transitioning from iPSC models to clinical treatments.	Facilitated exploration of enzyme replacement and other supportive therapies in a controlled setting.	Translating laboratory successes into viable clinical therapies remains slow and complex.	[13,18,39,43,44]

## 4. CRISPR-Cas9 Gene-Editing Technology for A1AD Disease Modeling

CRISPR-Cas9 is a groundbreaking genome-editing platform that has revolutionized the field of genetics by enabling precise modifications at specific locations in the genome of virtually any organism. Derived from a naturally occurring genome defense mechanism in bacteria, this technology utilizes a synthetic guide RNA (gRNA) to direct the Cas9 nuclease to a specific DNA sequence. The Cas9 enzyme then introduces a double-stranded break at the targeted location, which can be manipulated to either disrupt a gene, correct a mutation, or insert new genetic material [45]. This mechanism leverages the cell’s inherent DNA repair processes, primarily through non-homologous end joining (NHEJ) or homology-directed repair (HDR), to achieve the desired genetic alteration. NHEJ can introduce small deletions or insertions that disrupt gene function, which is helpful for gene knockout studies. In contrast, HDR, used in the presence of a donor DNA template, allows for precise gene correction or insertion, ideal for therapeutic applications [6,45].

Another advanced technology in genome editing is the development of variant Cas9 proteins that permit single-base editing and avoid double-strand break (DSB) events. These variant Cas9 proteins include Cas9 nickase (nCas9), which produces a single-stranded break rather than a DSB, or catalytically inactive Cas9 proteins such as dCas9, dCas12a, or dCas13b, enabling editing without DSBs [25]. The base-editing technology contains a cytosine base editor tool for converting C–G to T–A and an adenine base editor tool for shifting A–T to G–C. These are deaminases linked to a variant Cas9 and gRNA to modify a base pair at a specific locus [26]. With CRISPR base-editing technology, host DSB repair by either NHEJ or HDR is avoided and this allows the editing of a single base precisely without any errors created during DSB repair. Werder et al. employed adenine base editors (mediating A → G) to correct the G-to-A point mutation in the *SERPINA1* gene in patient-derived iPSCs. After differentiation of the edited iPSCs to hepatocytes, these derived hepatocytes reduced the aggregation of misfolded A1AT [27]. Their study showcased how precise genomic editing could correct the point mutation responsible for A1AT misfolding directly in the iPSCs from A1AD patients. This genetic correction led to a significant decrease in polymerized A1AT within the hepatocytes, suggesting a promising direction for gene therapy in A1AD [27]. Importantly, this study provided proof of concept that targeted gene editing could ameliorate disease phenotypes in patient-derived cellular models.

The targeted correction of pathogenic mutations within the *SERPINA1* gene using CRISPR-Cas9 technology represents a significant advancement in the treatment of A1AD. The *SERPINA1* gene encodes the alpha-1 antitrypsin protein, protecting the lungs from neutrophil elastase damage. Mutations in this gene, such as the PiZ allele, lead to the production of a misfolded alpha-1 antitrypsin protein that accumulates in hepatocytes and significantly reduces its levels in the bloodstream, contributing to lung and liver diseases [46]. The PiZ allele is characterized by a single point mutation (Glu342Lys) that results in a polymerization-prone protein [47]. Successful instances of gene editing have been documented where iPSCs derived from patients with A1AD were used as models to perform precise genetic interventions aimed at correcting the PiZ allele [48]. Researchers have effectively replaced the mutated segment with a healthy copy of the gene by designing gRNA to target this specific mutation and using HDR with a correct DNA template. This correction restores the normal folding and function of the alpha-1 antitrypsin protein [27].

**Figure 1:**
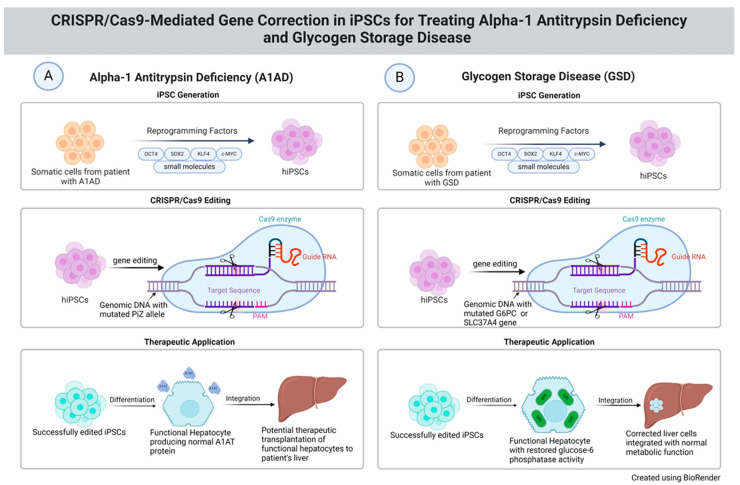
Gene editing using CRISPR-Cas9 in iPSCs for therapeutic gene corrections associated with alpha-1 antitrypsin deficiency and glycogen storage disease.

As illustrated in Figure 1, the CRISPR-Cas9 system was employed to specifically target and correct the PiZ allele mutations in the *SERPINA1* gene. The gRNA was designed to align with the mutation site precisely, directing the Cas9 nuclease to induce a DSB at this specific locus within the genome. The use of HDR mechanisms was crucial, as it allowed for the introduction of the correct SERPINA gene sequence via a donor DNA template, effectively replacing the mutated segment with a healthy copy of the gene [10,39]. Post editing, the iPSCs showed a restoration of functional A1AT protein levels. The edited iPSCs, differentiated into hepatocyte-like cells, demonstrated the ability to produce A1AT at levels comparable to those observed in healthy individuals. In another study, as a dominant mutation in patients is PiZ *SERPINA1* carrying a single G → A mutation, by using an advanced adenine base editor tool in base-editing technology, researchers were able to correct the mutated single base pair from G⋅C to A⋅T [46]. In vivo experiments with PiZ-transgenic mice demonstrated that these cells exhibited reduced aggregation of the misfolded protein, a critical factor in preventing the hepatocyte and lung damage typically associated with A1AD. This outcome not only signifies a direct therapeutic potential but also enhances our understanding of the disease’s pathophysiology, providing invaluable insights into the mechanisms underlying A1AD and its treatment [46].

The success of these gene-editing endeavors opens potential pathways for developing targeted gene therapies that could be personalized based on a patient’s genetic profile. Such therapies could potentially offer a permanent solution to A1AD, moving beyond symptomatic treatment to address the root cause of the disease. As we advance our capabilities in gene editing, further refinements in CRISPR-Cas9 technology, such as the development of high-fidelity Cas9 enzymes, are anticipated. These advancements are expected to enhance the precision and reduce off-target effects, significantly improving the safety profile of gene therapies for clinical use [6].

Despite these promising results, several challenges remain. The variability of iPSC lines due to differences in the genetic background of the donor cells and the reprogramming process can affect the consistency of disease models and therapeutic outcomes. Furthermore, ensuring the long-term stability and integration of corrected genes without off-target effects remains a significant hurdle. These technical and biological challenges necessitate ongoing research and development, as well as comprehensive preclinical and clinical trials to establish the efficacy and safety of the treatments before they can be widely implemented [12].

## 5. CRISPR-Cas9 Gene-Editing Technology for Modeling GSDI 

Gene editing has emerged as a cornerstone in the therapeutic landscape for GSDI to correct mutations in the *G6PC* gene for type Ia and the *SLC37A4* gene for type Ib. The gRNA is meticulously designed to pair with the exact sequence of these mutations, ensuring the Cas9 enzyme introduces DSBs precisely at the faulty gene sites. This targeted approach utilizes HDR mechanisms to introduce a correct copy of the gene via a donor template, effectively replacing the defective segment with a healthy version. This correction restores the function of the glucose-6-phosphatase enzyme, which is crucial for the proper metabolic processing of glycogen into glucose [37,38]. Arnaoutova and colleagues have demonstrated in vivo correction in a mouse model using CRISPR-Cas9 to specifically target and correct the *G6PC-p*. R83C mutation, a prevalent pathogenic variant. The correction of this mutation restored glucose-6-phosphatase activity in liver cells, enabling these cells to manage glycogen breakdown and glucose release effectively, thus reducing glycogen accumulation and the associated organ damage [38]. Furthermore, Skakic et al. reported on using CRISPR-Cas9 in iPSCs derived from GSDIb patients. These iPSCs were edited to correct the *G6PC* mutation and differentiated into hepatocyte-like cells. The edited cells exhibited restored functionality of glucose-6-phosphatase, as evidenced by their ability to perform gluconeogenesis and glycogenolysis effectively. Post-editing metabolic assays confirmed the normalized metabolic pathways in these cells, alongside their ability to maintain stable blood glucose levels under fasting conditions [49]. Rutten and colleagues generated a hepatocytic GSDIa mouse model via CRISPR-Cas9 and characterized the effects of G6PC on glucose metabolism and liver functions [50]. Their study revealed that genome-edited mouse models allow for the modeling of a variety of GSDIa phenotypes [50]. Rossiaud and colleagues expanded on this work by highlighting the role of iPSC technology in modeling disease pathology and testing gene therapy approaches in vitro. They detailed how genetically corrected hepatocytes derived from iPSCs of GSDIII patients could be used to study the disease mechanisms and the efficacy of new gene therapy protocols in a controlled environment, thus providing valuable insights before clinical application [19].

These advancements in gene editing for GSDI open new avenues for treatment beyond dietary management and symptomatic care. By addressing the root cause of metabolic dysfunctions, gene therapy offers a potential for lasting remedies that could significantly enhance the quality of life and reduce the burden of disease management. As gene-editing techniques evolve, particularly with advances in delivery mechanisms and editing efficiency, the prospect of applying these therapies in clinical settings becomes increasingly feasible. Future clinical trials will be crucial in evaluating the safety, efficacy, and long-term benefits of these treatments [43,50].

Despite these advancements, the application of gene editing in GSDI faces several challenges. Variability in gene correction efficiency across different iPSC lines and the potential for off-target effects remain significant concerns. Ensuring the precision and stability of gene edits is paramount to prevent unintended consequences and guarantee therapeutic efficacy. Furthermore, the scalability of these technologies for widespread clinical use requires ongoing innovation and rigorous regulatory scrutiny to ensure that treatments can be delivered safely and effectively [49,51].

## 6. Technical Challenges in Disease Modeling and Gene Therapy

The complexity of disease phenotypes hinders the accurate modeling of diseases via editing genes. Modeling diseases using iPSCs often encounters the complexity of replicating the multifaceted nature of diseases, especially those with complex genetic and environmental interactions. Diseases like A1AD and GSDI involve multiple organ systems and biochemical pathways, which can be challenging to replicate in iPSC-derived models accurately. This complexity can lead to models that do not fully capture the disease phenotype or its progression [52]. On the other hand, while CRISPR-Cas9 offers revolutionary precision in gene editing, several technical challenges persist. Off-target effects, where CRISPR-Cas9 inadvertently edits genes other than the intended targets, can lead to unintended consequences and complicate the interpretation of experimental results. Furthermore, the site-specific editing efficiency, crucial for precise gene correction, is naturally low in many cell types, which limits the applications of corrective gene editing [6,45,53]. Furthermore, variability in iPSC lines, due to differences in the genetic background of the donor cells and the reprogramming process, can affect the consistency of disease models and therapeutic outcomes. Moreover, scaling up iPSC technologies for clinical applications poses significant challenges, including maintaining cell quality and ensuring standardized protocols across different batches of cells [12].

## 7. Ethical Dilemmas and Regulatory Landscape of Using iPSCs and CRISPR-Cas9 

Using iPSC and CRISPR-Cas9 technologies raises several ethical issues, particularly concerning germline editing, which can affect future generations. The potential for creating “designer babies” with selected traits has sparked intense ethical debates. Moreover, the use of CRISPR-Cas9 in embryos and reproductive cells is subject to stringent ethical scrutiny to prevent possible misuse [44]. However, these ethical issues are of less concern in the treatment of these debilitating genetic diseases. Regulatory frameworks for gene-editing therapies are still in development, with significant variations between countries. The approval process for therapies developed using CRISPR-Cas9 and iPSCs can be lengthy and complex, involving multiple stages of clinical trials to ensure safety and efficacy. Regulatory agencies like the Food and Drug Administration (FDA) and the European Medicines Agency (EMA) are actively working to establish guidelines that address both the scientific aspects of these therapies and their ethical implications [54]. In addition, using human cells for iPSC generation involves considerations regarding consent and privacy. Donors must be fully informed about how their cells will be used, the potential for commercialization, and the privacy measures taken to protect their genetic information. Ensuring transparent consent processes is crucial for ethical compliance and public trust [51].

## 8. Future Directions

Integrating CRISPR-Cas9 genome-editing technologies with iPSC platforms heralds a transformative era in precision medicine. This shows extraordinary potential for the treatment of metabolic and genetic disorders such as GSDI and A1AD. CRISPR-Cas9’s precision in targeting specific genetic anomalies presents a promising avenue for directly addressing the genetic bases of these diseases, while iPSCs facilitate the development of patient-specific organoid models, revolutionizing drug screening and disease modeling. Recent advancements such as the development of liver organoids from iPSCs that accurately mimic the disease phenotype of A1AD demonstrate the potential of these technologies to revolutionize therapeutic strategies [17,31,55]. Moreover, CRISPR-Cas9 has been utilized to correct mutations directly associated with A1AD or GSDI in patient-derived iPSCs, setting the stage for these cells to be differentiated into functional tissue for transplantation without the ethical and immunological complications associated with traditional stem cell therapies [34,50,56].

Despite these advancements, the clinical application of these technologies faces significant challenges, primarily concerning safe and effective delivery mechanisms. Research into various vectors for CRISPR-Cas9 delivery has underscored the need for a balance between efficacy and safety, particularly in reducing off-target effects that could lead to unintended genetic consequences [51,57]. Similarly, the complexity of mimicking exact physiological conditions within iPSC-derived organoids remains a significant hurdle, crucial for accurate disease modeling and therapeutic development [58]. Furthermore, the path from laboratory models to clinical applications involves intricate technological, regulatory, and ethical considerations. Rigorous clinical trials are needed to validate the efficacy and safety of these technologies. Additionally, comprehensive regulatory frameworks and ethical guidelines must be refined to ensure these innovations translate safely from the test bench to the bedside. This includes addressing potential issues related to genetic privacy, germline modifications, and the implications of creating genetically altered human cells. Additionally, interdisciplinary collaborations across fields such as bioinformatics, genetic counseling, and regulatory sciences will be essential in addressing these challenges. Such collaborations can lead to innovative solutions that harness the full potential of iPSC and CRISPR-Cas9 technologies, facilitating their transition into mainstream clinical practices.

Overall, the integration of CRISPR-Cas9 and iPSC technologies holds tremendous promise for treating diseases like GSDI and A1AD. However, the certainty of these future directions is hindered by significant obstacles that must be overcome. Through the concerted efforts of scientists, clinicians, ethicists, and policymakers, the full potential of these groundbreaking technologies can be realized, leading to more effective and personalized therapeutic options for patients with genetic and metabolic disorders.

## 9. Conclusions

The integration of iPSC and CRISPR-Cas9 technologies has revolutionized the field of biomedical research, particularly in the study and treatment of complex and rare metabolic and genetic disorders such as A1AD and GSDI. This review has highlighted significant advancements in and the transformative potential of these technologies for modeling diseases, correcting genetic anomalies, and paving the way for innovative therapeutic solutions. iPSC technology has enhanced our ability to model diseases accurately and tailor treatments to individual genetic profiles. These models provide crucial insights into disease pathophysiology and assist in optimizing pharmacological therapies. Similarly, CRISPR-Cas9 offers unprecedented precision in gene editing, providing robust frameworks for studying and directly correcting genetic defects at their source.

However, these advancements come with significant challenges. Technical issues such as ensuring the fidelity and safety of gene edits, overcoming the variability of iPSC lines, and scaling up for clinical applications are formidable. Moreover, ethical and regulatory considerations must be meticulously navigated to ensure patient safety, particularly concerning potential off-target effects and germline editing implications. The path forward will require continued innovation and an interdisciplinary approach to overcome these hurdles. Enhancing the precision of gene editing, improving the efficiency of iPSC derivation and differentiation, and addressing regulatory and ethical challenges will be crucial. This collaborative effort across scientific, regulatory, and ethical domains is essential for realizing the full potential of these technologies in treating A1AD, GSDI, and other genetic disorders.

In conclusion, while significant obstacles remain, the potential of iPSCs and CRISPR-Cas9 to fundamentally transform the landscape of disease modeling and gene therapy is immense. The ongoing research and development are not merely academic exercises but are paving the way for future therapeutic breakthroughs that could revolutionize medicine.

## Figures and Tables

**Table 1:** Comparative analysis of the pathophysiologies and clinical presentations of A1AD and GSDI.

Feature	Alpha-1 Antitrypsin Deficiency (A1AD)	Glycogen Storage Disease Type I (GSDI)
Genetic Basis	Mutation in the *SERPINA1* gene leads to a defective production of alpha-1 antitrypsin.	Mutations in the G6PC (GSDIa) or *SLC37A4* (GSDIb) genes affect glucose-6-phosphatase.
Pathophysiology	Accumulation of misfolded A1AT protein in the liver, impairing its release into the bloodstream and reducing its protease inhibitor activity.	Deficient activity of glucose-6-phosphatase disrupts glycogenolysis and gluconeogenesis, causing excessive glycogen and fat accumulation in the liver and kidneys.
Primary Organ Impact	Liver and lungs.	Liver and kidneys.
Clinical Manifestations	Pulmonary disorders such as early-onset emphysema and COPD; liver disease, ranging from mild enzyme elevations to cirrhosis; and hepatocellular carcinoma.	Severe hypoglycemia, hepatomegaly, growth retardation, metabolic acidosis, and progressive renal disease.
Common Symptoms	Shortness of breath, wheezing, and liver dysfunction.	Hypoglycemia symptoms (e.g., fatigue, irritability), enlarged liver, stunted growth.
Treatment Approaches	Augmentation therapy (infusion of A1AT) and liver transplantation in severe cases.	Dietary management (frequent carbohydrate-rich meals), medications to control metabolic symptoms, liver transplantation in severe cases.
Prognosis	Variable: depends on the degree of lung and liver disease. Life expectancy can be near normal with appropriate management.	Chronic and managed conditions; complications like kidney disease can impact life expectancy if not properly managed.

## Data Availability

Not applicable.
